# Random nanohole arrays and its application to crystalline Si thin foils produced by proton induced exfoliation for solar cells

**DOI:** 10.1038/s41598-019-56210-7

**Published:** 2019-12-24

**Authors:** Hyeon-Seung Lee, Jae Myeong Choi, Beomsic Jung, Joonkon Kim, Jonghan Song, Doo Seok Jeong, Jong-Keuk Park, Won Mok Kim, Doh-Kwon Lee, Taek Sung Lee, Wook Seong Lee, Kyeong-Seok Lee, Byeong-Kwon Ju, Inho Kim

**Affiliations:** 1 Center for Electronic Materials, Korea Institute of Science and Technology, Seongbuk-gu, Seoul 02792 Republic of Korea; 2 School of Electrical Engineering, Korea University, Seoul, 02841 Republic of Korea; 3 Advanced Analysis Center, Korea Institute of Science and Technology, Seongbuk-gu, Seoul 02792 Republic of Korea; 4 Division of Materials Science and Engineering, Hanyang University, Seoul, 04763 Republic of Korea; 5 Photo-electronic Hybrids Research Center, Korea Institute of Science and Technology, Seongbuk-gu, Seoul 02792 Republic of Korea; 6 Present Address: Hanwha Q CELLS Korea Corporation, Chungcheongbuk-do, 27816 Republic of Korea

**Keywords:** Devices for energy harvesting, Electronic properties and materials

## Abstract

We report high efficiency cell processing technologies for the ultra-thin Si solar cells based on crystalline Si thin foils (below a 50 µm thickness) produced by the proton implant exfoliation (PIE) technique. Shallow textures of submicrometer scale is essential for effective light trapping in crystalline Si thin foil based solar cells. In this study, we report the fabrication process of random Si nanohole arrays of ellipsoids by a facile way using low melting point metal nanoparticles of indium which were vacuum-deposited and dewetted spontaneously at room temperature. Combination of dry and wet etch processes with indium nanoparticles as etch masks enables the fabrication of random Si nanohole arrays of an ellipsoidal shape. The optimized etching processes led to effective light trapping nanostructures comparable to conventional micro-pyramids. We also developed the laser fired contact (LFC) process especially suitable for crystalline Si thin foil based PERC solar cells. The laser processing parameters were optimized to obtain a shallow LFC contact in conjunction with a low contact resistance. Lastly, we applied the random Si nanohole arrays and the LFC process to the crystalline Si thin foils (a 48 µm thickness) produced by the PIE technique and achieved the best efficiency of 17.1% while the planar PERC solar cell without the Si nanohole arrays exhibit 15.6%. Also, we demonstrate the ultra-thin wafer is bendable to have a 16 mm critical bending radius.

## Introduction

The use of thinner wafers is one of the most straightforward methods to lower the module price of the crystalline Si solar cells because the cost of the Si material account for more than 30% of the module^1^. The incessant research efforts have been made to develop the fabrication techniques to produce the thinner Si wafers. Currently, a multi-wire sawing has been adopted for Si wafer fabrication by the photovoltaics industry; however, this technique will face the wafer thickness limitation in the near term future due to the finite wire size making it difficult to produce the Si wafers thinner than 80 μm^2^.

Several techniques such as proton induced exfoliation^3^, metallic stressor induced spalling^4,5^, electrodeposit-assisted stripping (EAS)^6^ and epitaxial lift-off^7^ have been proposed for kerfless wafering of thin Si wafers or thin foils (<50 µm) to reduce a Si material loss in the conventional wafering method to lower the module cost. Proton induced exfoliation (PIE) which we adopted in this study is one of the promising kerfless techniques due to the process simplicity of implantation and cleaving. In this technique, protons are implanted into Si donor wafers with MeV acceleration energy. In the subsequent thermal treatment, the implanted protons aggregate and turn into hydrogen gas, which induces the crack propagation resulting in the cleavage of the thin Si wafers. However, the efficiency of the solar cells based on the kerfless thin wafer fabricated by proton induced exfoliation has been reported to lag behind the counter part technology based solar cells. The epitaxial lift-off solar cells have reached an efficiency of 21.2%^8^, and the metallic stressor induced spalling solar cells showed an efficiency of 14.9%^9^ whereas the thin Si wafers produced by the PIE process only led to 13.2% with a standard cell architecture of Al back surface field and recently reached 15.2%^3,10^.

One of the main reasons for the lower efficiency of the PIE solar cells arises from a difficulty in texturing. The critical proton dose for the exfoliation of the Si kerfless inherently relies on the Si crystal orientation. The (111) orientation known as a cleavage plane has the lowest the threshold proton dose for exfoliation^11,12^. However, for the application of the (111) thin wafers to high efficiency solar cells, it is necessary to cope with texturing of the (111) wafers for effective light trapping. The conventional pyramid texturing with alkaline solution is not applicable to the Si wafers of a (111) orientation because the etch rate of the (111) surface is extremely slower compared with the (100) one^13^. In our previous report, we combined laser interference lithography and a reactive ion etch process for nano-scale texturing of the kerfless-thin wafers with a (111) crystal orientation^10^. However, the laser interference lithography has a limitation in the large area process^14^. In this study, we developed an isotropic nano-texturing process with a low melting point metal as etch mask which can be processed in the large area. We demonstrate that our nano-texturing provides high light trapping performances comparable to conventional micro-pyramid textures. Many interesting approaches to fabricate the semiconductor nanostructures of various shapes have been reported and demonstrated to show performance boost up of the optoelectronic devices such as solar cells, photodetectors and light emitting diodes^15–19^. Further improvements of optical performances would be expected by introducing the novel three dimensional nanostructures in our ultrathin Si solar cells.

For the successful adoption of the Si thin foils in the photovoltaic industry, the cell processing technology of metallization especially designed for the thin foils need to be developed. The conventional metallization process based on screen printing using thick metal pastes is hardly applicable to the thin Si foils because of the severe wafer bowing induced by the thermal expansion coefficient differences between metal electrodes and Si wafers especially at high temperature in the range of 700 °C^20^. This can be avoided by metallization at low temperature. Laser metallization using pulse laser in nanosecond can be one of the viable options for the bowing free metallization. We adopted a PERC cell for the thin Si foil based solar cells. The formation of the Al local back surface (LBSF) is required for the PERC cell, and the pulse laser incident into aluminum at the rear side of the PERC cells locally melts the Al electrode, which penetrate into the passivation layers for Al LBSF formation. This LBSF formation process using the pulse laser is termed as laser fired contact (LFC) process coined by the German research group^21^. In this study, we adapted the LFC process into the metallization process compatible with the thin Si foil based solar cells by optimization of laser processing parameters.

The thin Si foils of a 58 µm thickness and a maximum size of 5 cm × 5 cm were prepared by the PIE technique. The kerfless thin Si foils were cleaved from the donor wafers of a (111) crystal orientation by implantation of 2.3 MeV proton beams and subsequent annealing. Si random nanohole array textures were fabricated by two step processes of wet and dry etching with indium metallic islands as etch masks. The width and depth of the nanohole structures were simply adjusted by varying the size of the indium islands and the etching process time. We introduced the random nanohole array textures on the kerfless thin Si foils of a 48 µm thickness and by combining the LFC process, demonstrating an efficiency of 17.1%, which is the best efficiency among the solar cells based on the kerfless thin wafers produced by the PIE technique to the best of our knowledge.

## Results

### Kerfless wafering

**Figure 1:**
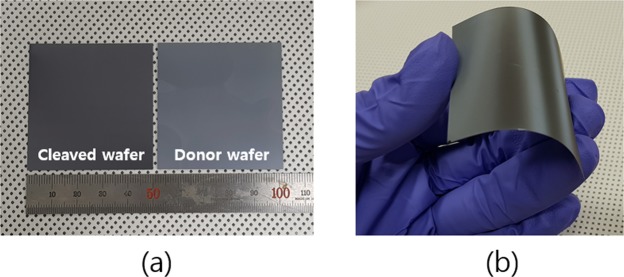
(**a**) Crystalline Si thin foils of a 58 µm thickness as-cleaved by the proton induced exfoliation (PIE) technique. (**b**) Demonstration of the flexible Si thin foil.

For the fabrication of the kerfless Si thin wafers by the PIE technique, the proton beams of 2.3 MeV energy are implanted into the donor wafer of a (111) crystal orientation. Subsequently, the donor wafer is annealed for hydrogen micro bubble formation and crack propagation for the kerfless wafer cleavage^3^. The donor wafer can be reused in multiple times for the kerfless wafering^10^. The proton beams are implanted by raster scan to have a uniform dose of 1 × 10^17^ cm^−2^. The cleaved thin foil of a 58 µm thickness and a 5 cm × 5 cm size was produced and shown together with a donor wafer in Fig. 1(a). It was reported that the planar Si foil of a 50 µm thickness is bendable to have a bending radius of 37 mm owing to its thin thickness^22^. The bending of the cleaved Si thin foil is demonstrated and shown in Fig. 1(b). The implantation damage at the cleaved surface of the kerfless Si thin foils was completely removed by isotropic etching of ~5 µm thickness at both of the front and rear surfaces before the cell processing. The thickness of the thin Si foils was measured to be 48 µm by scanning electron microscopy (SEM) after the damage removal etching. Our fabrication procedure of the kerfless Si thin foils by the PIE technique and the damage removal process were more detailed in our previous publication^10^.

### Random nanohole arrays texturing

**Figure 2:**
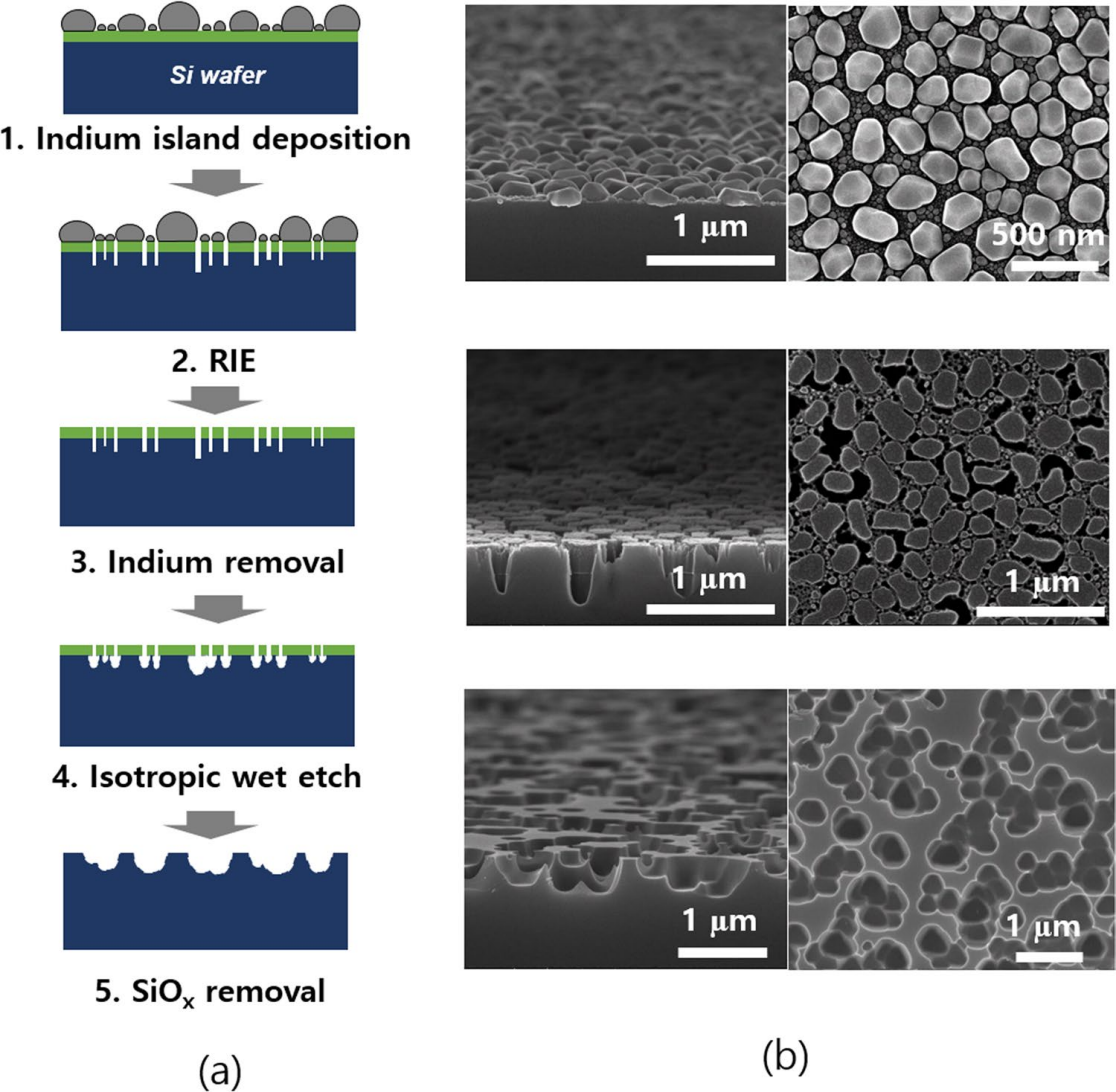
(**a**) Process flow schematics of random Si nanohole structures with indium islands. (**b**) SEM images the Si wafers corresponding step 1 (top), step 3 (middle), and step 5 (bottom) in the in tilted and plan views.

The thin Si foils were cleaved from the donor wafers of a (111) crystal orientation. The conventional pyramid texturing in micro scale by alkaline solutions is not applicable to the (111) wafers because the etch rate of the (111) Si surface in the alkaline solutions is known to be extremely slow^13^. On the other hand, the light trapping becomes more challenged in the thinner Si foils. Thus, the loss of the Si material during texturing needs to be reduced, and it is more desirable that the texture scale is sub-micron scale. For these reasons, we developed a nano scale isotropic texturing process which is applicable to the thin c-Si foils of a (111) orientation in large area. The texture process we propose used two step dry and wet etching processes with low melting point etch masks. The overall texture process is presented in Fig. 2(a). A low melting point of indium is thermally evaporated onto the SiO_x_ coated Si wafers. The indium metal exhibits island growth mode known as Volmer-Weber mode^23^. The size of the indium metal islands can be adjusted by varying the indium nominal thickness. The isolated indium islands in nano scale serve as the etch masks for the random nanohole arrays texturing. Interestingly, the low melting point metals are known to grow in a bimodal size distribution. The large size metal islands are dominantly observed, and the small size islands grow nearby the large islands.

As a second step, reactive ion etch is performed with a CF_4_/O_2_ mixed gas to etch the SiO_x_ layer in a high anisotropy. As a result, the Si nano pillars in a bimodal size distribution are obtained. The metallic indium islands are selectively removed by a HCl solution. In the next step, the Si wafer is further etched with an isotropic etchant of HF:HNO_3_:H_2_O with the SiO_x_ as etch masks. In this step, the small size Si nano pillars are selectively etched and turns into the larger holes. Lastly, the SiO_x_ layer is removed, and the random nanohole structures are obtained. Figure 2(b) show the cross-sectional scanning electron microscopy (SEM) images corresponding to step 1, 4, and 5 in Fig. 2. In this case, the nominal thickness of the indium metal was 50 nm. Note that the small size Si pillars in Fig. 2(b) are etched and combined into the larger nanohole structures as seen in the SEM images at the bottom corresponding to step 5. Thus, the maximum size of the Si nanoholes are dominantly influenced by the particle to particle distance as illustrated in Fig. 2(a).

**Figure 3:**
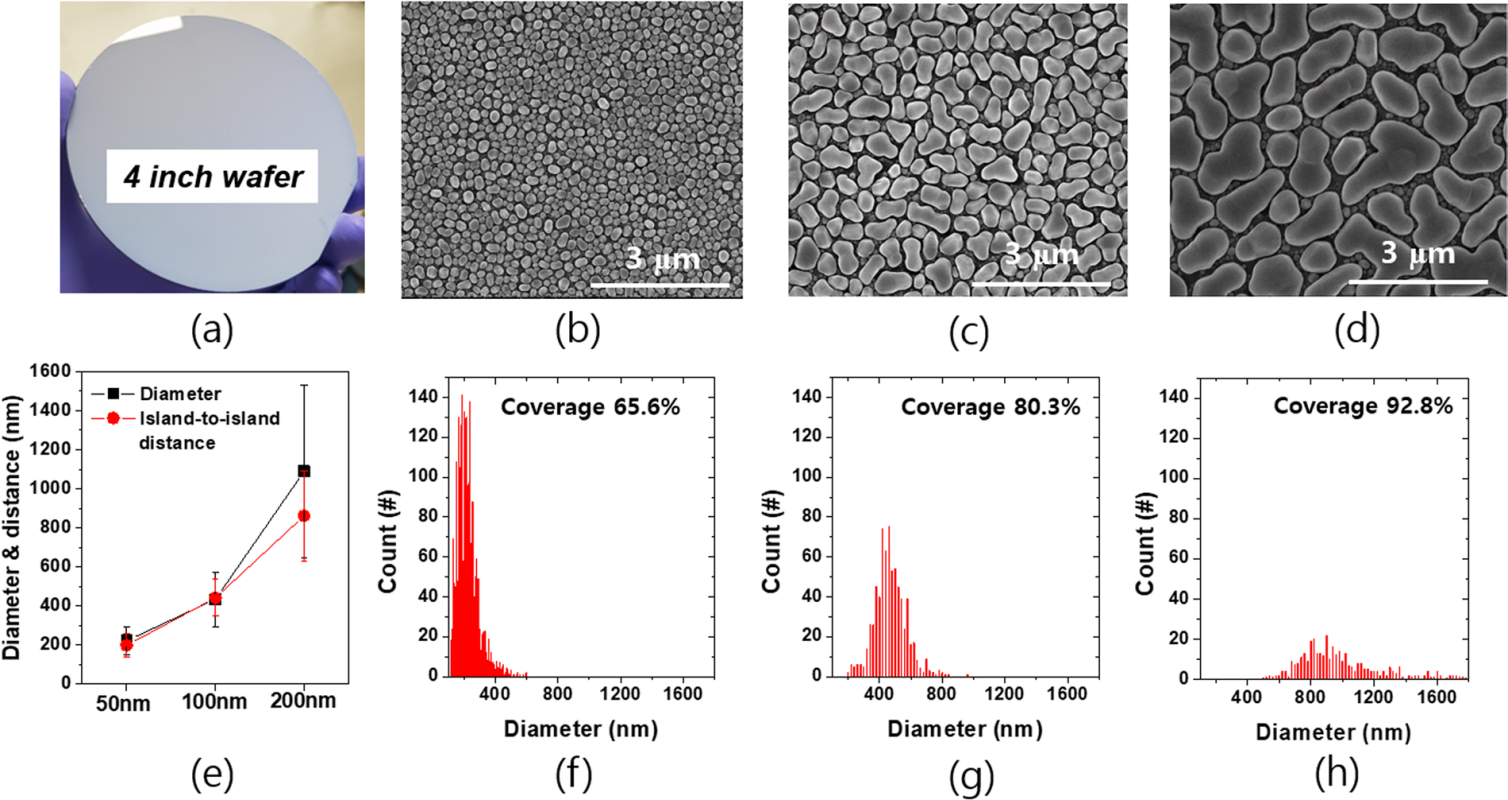
(**a**) Photograph image of a 4 inch Si wafer with indium islands of a 50 nm nominal thickness. SEM images of indium islands on Si wafers. The nominal thicknesses of indium varies for (**b**) 50 nm, (**c**) 100 nm, and (**d**) 200 nm. (**e**) Average indium particle diameters and distances of nearest neighboring particles for various indium nominal thicknesses. (**f**–**h**) Size distributions and areal coverages of indium nanoparticles with varying nominal thicknesses.

Our proposed scheme of nano texturing can be applicable to large area wafers by combining uniform deposition of the metal islands and etching. The indium islands of a 50 nm nominal thickness, which were evaporated onto a 4 inch wafer without intentional heating of substrates, is shown in Fig. 3(a) as an example. The indium island etch masks can be adjusted in a facile way by varying the nominal thickness of the indium metal. The indium islands with varying the nominal thickness from 50 nm to 200 nm were observed by SEM as shown in Fig. 3(b–d). As the nominal thickness increases, the shape of the indium islands becomes more irregular and the size increases linearly. The size distribution, average diameter and average island-to-island distance were analyzed using an image processing software (Image J) and presented in Fig. 3(e–h). For convenience, the smaller particles than the half of the average diameter were excluded for the analysis, and the diameter was extracted assuming the shape of indium islands is circular. The average island-to-island distance was determined by measuring the distance between the center points of the adjacent islands. The fabrication techniques of the Si nanostructure formation using the dewetted metal etch masks are well known but mostly the noble metals such as Ag, Au and Cu have been used^24–27^. However, those novel metal elements have relatively high melting point (T_m_); thus, post heat-treatment is prerequisite for formation of a few hundred nanometer size islands. The homologous temperatures (T/T_m_) for the above noble metals at room temperature are all in the range of 0.22~0.24, whereas indium has much higher T/T_m_ of 0.69 which enables spontaneous dewetting even without substrate heating. The island size of typical noble metals increases much faster than the nominal thickness following the relationship that the average particle size (d_avg_) increases with the nearly square of the nominal thickness (t_metal_) of the metals: d_avg_ ~ t_metal_^5/3^ ^28^. The coverage of the dewetted noble metals is in the range of 30 ~ 40%. The sensitive increase with the metal thickness and the low coverage make it challenging to apply the noble metal dewetting to the fabrication of the effective light trapping nano structures. In contrast, the growth behavior of the indium metal is quite different in that the average particle size increases linearly with the nominal thickness (d_avg_ ~ t_metal_) and the coverage of the metal islands is greater than 60% as presented in Fig. 3. The less sensitive increase of the particle size with the nominal thickness provides a wider process window for nano structure fabrication, and the high coverage leads to more effective light trapping.

### Light trapping performances of the random Si nanoholes

**Figure 4:**
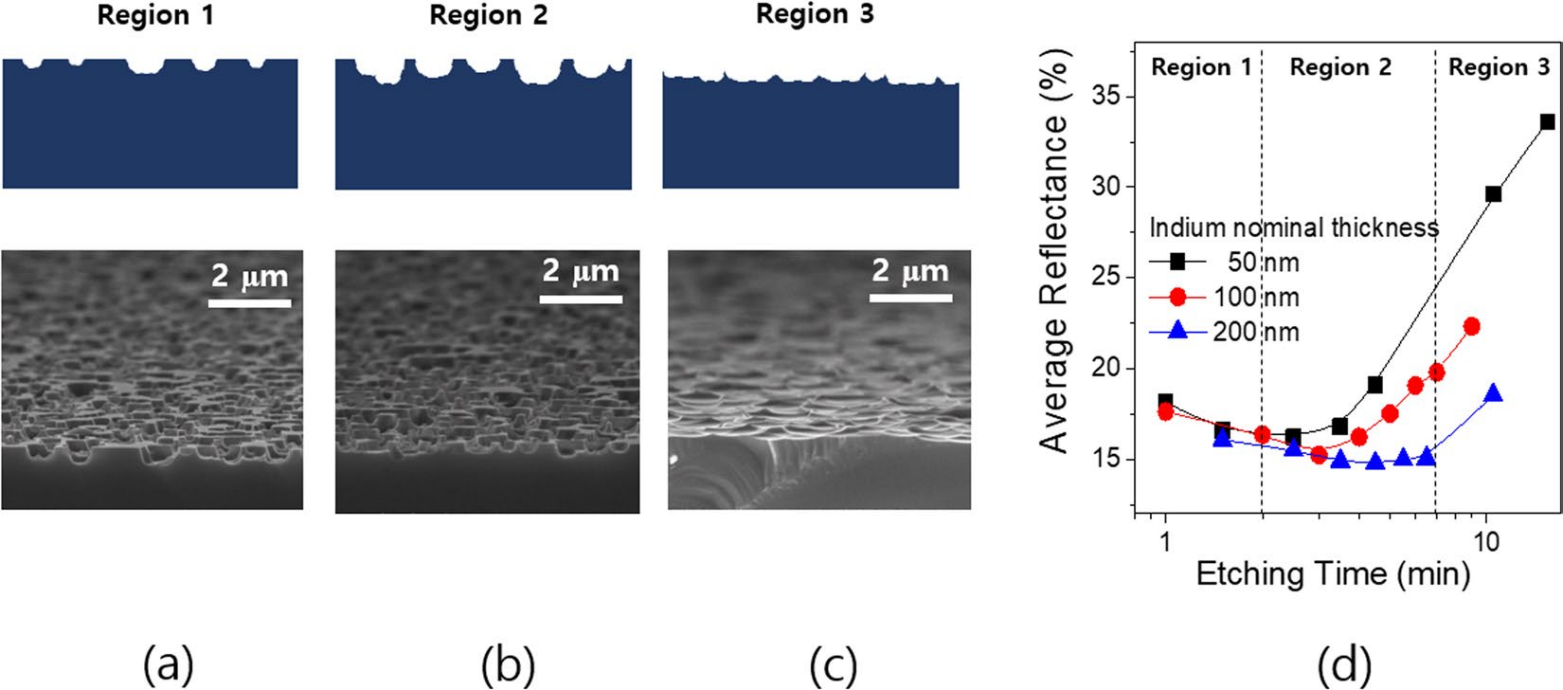
(**a**–**c**) Cross-sectional schematics and SEM images of the random Si nanohole structures with varying the isotropic etch time. Region 1, 2 and 3 correspond to the early etch stage, the optimal etch stage and the late etch stage, respectively. (**d**) Solar weighted average reflectances with varying the indium nominal thickness and the etch time. The optically effective nanostructures are formed at region 2.

The geometrical factors of the Si nanohole structures such as width and depth are crucial for effective light trapping^29^. The shape of the Si nano structures in the isotropic wet etch step is determined by the etch process time. The nanohole textures we developed are classified into three different regions depending on the process time as illustrated together with the corresponding SEM images in Fig. 4. In the early stage (region 1), the shallow Si nanoholes are formed by the isotropic etchant but a major part of the surface is flat yet. In the second stage (region 2), the Si nano hole structures become deeper and the flat regions are reduced with the increasing the etch process time. In the last stage (region 3), the flat regions almost disappear and the nanohole structures are hardly observed. The SEM images of the wafers textured with indium islands of a 50 nm nominal thickness at each stage are presented in Fig. 4(a–c). The total reflectances of the textured Si wafers with the etching time for the different indium thicknesses were measured and shown in Fig. 4(d). In the early stage, the reflectance of all the wafers decreases with increasing the etch process time. The textured wafers exhibit the minimum reflectances in region 2, and the reflectances of all the wafers increase with further increasing the etching time in region 3. Note that the thicker indium etch mask provides the lower minimum reflectance with the longer etch time. This is because the thicker indium leads to the larger nanohole structures which are more beneficial for light trapping. We performed the optical simulations to find the optimal design of the ellipsoidal nanohole structures (Fig. S1). The light trapping by the Si nanostructures can be understood by the Mie resonance combined with a graded index effect^30–32^. In general, the graded index effect is dominant in the longer wavelength range than the nanostructure size, whereas the Mie resonance becomes significant where the nanostructure sizes are smaller than the light wavelengths^33^. The Mie resonator on the high refractive index substrate such as Si strongly induce forward scattering of the incident light leading to the greatly enhanced light path length^34^. The simulation results reveal that the ellipsoidal nano holes in the rage of 500 ~ 750 nm provide stronger light trapping especially in a weakly absorbing range from 700 nm to 1200 nm which is beneficial for effective light trapping in thin Si foils.

**Figure 5:**
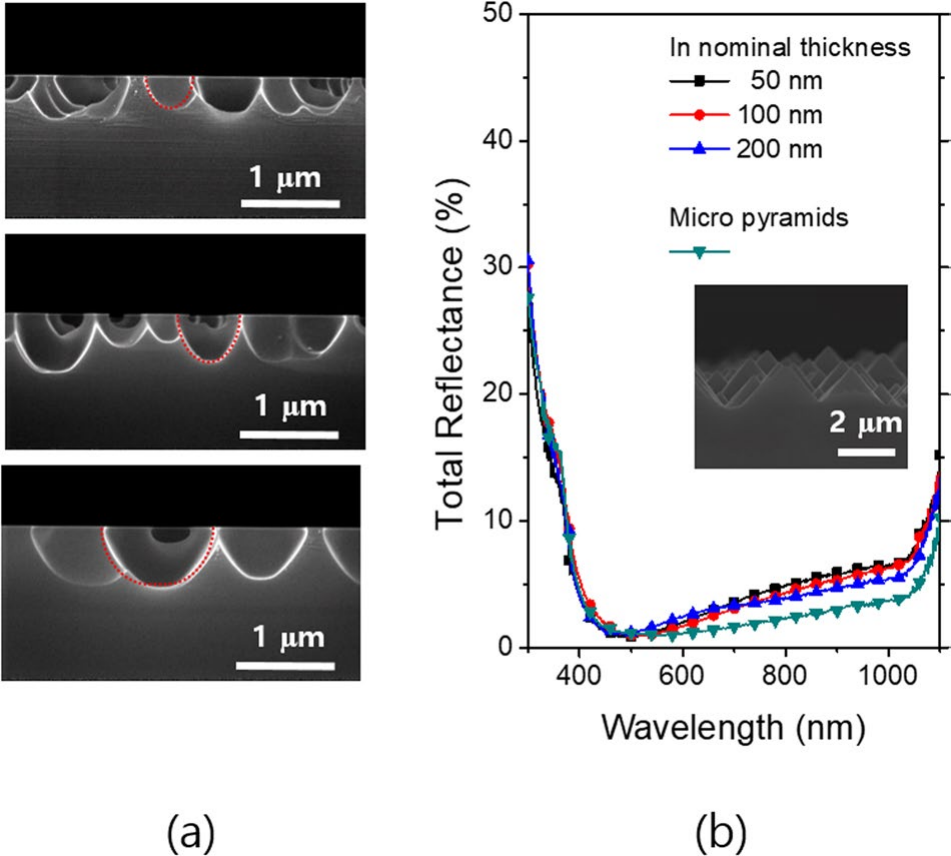
(**a**) Cross-sectional SEM images of the random Si nanohole arrays fabricated with different indium nominal thicknesses of 50 nm (top), 100 nm (middle) and 200 nm (bottom). Total reflectances (diffuse + specular) of the Si wafers (525 µm thickness) textured with the Si nanohole arrays and coated with a single layer antireflection coating of SiN_x_ (70 nm). The inset figure is the cross-sectional SEM image of the conventional pyramids in micrometer scale.

The largest nanohole structures are achievable in region 2, and the cross sectional SEM images taken in this stage for three different indium thicknesses of 50 nm, 100 nm and 200 nm are shown in Fig. 5(a). The nanohole shapes can fit in the ellipsoid denoted by the red dotted lines. The deeper nanoholes can be obtained with increasing the indium nominal thickness, and all the nanoholes are below in sub micrometer range. The average depths of the nanoholes for the indium nominal thicknesses of 50 nm, 100 nm, and 200 nm were 320 nm, 520 nm and 828 nm, respectively. The antireflection layer of SiN_x_ was deposited on the textured wafers showing the minimum reflectances in region 2, and the total reflectance spectra of the textured wafers were taken and presented in Fig. 5(b). For comparison, the total reflectance spectrum of the (100) wafers conventionally textured with micro scale pyramids is shown together. All the nanohole texture wafers have slightly higher reflectances especially in the long wavelength region above 600 nm in comparison with the micro scale pyramid texture but mostly they showed comparable antireflection performances. As shown in Fig. 4, the minimum reflectances of the nanohole textures become lower with increasing the indium thickness without the SiNx anti-reflection layer. However, the SiNx coated nanohole textures do not show significant differences as seen in Fig. 5. The thicker indium leads to the wider and deeper hole structures as shown in Fig. 5(b). In this study, we aimed at the shallow nanostructures in the depth. For this reason, we chose 50 nm of an indium thickness for the cell fabrication.

**Figure 6:**
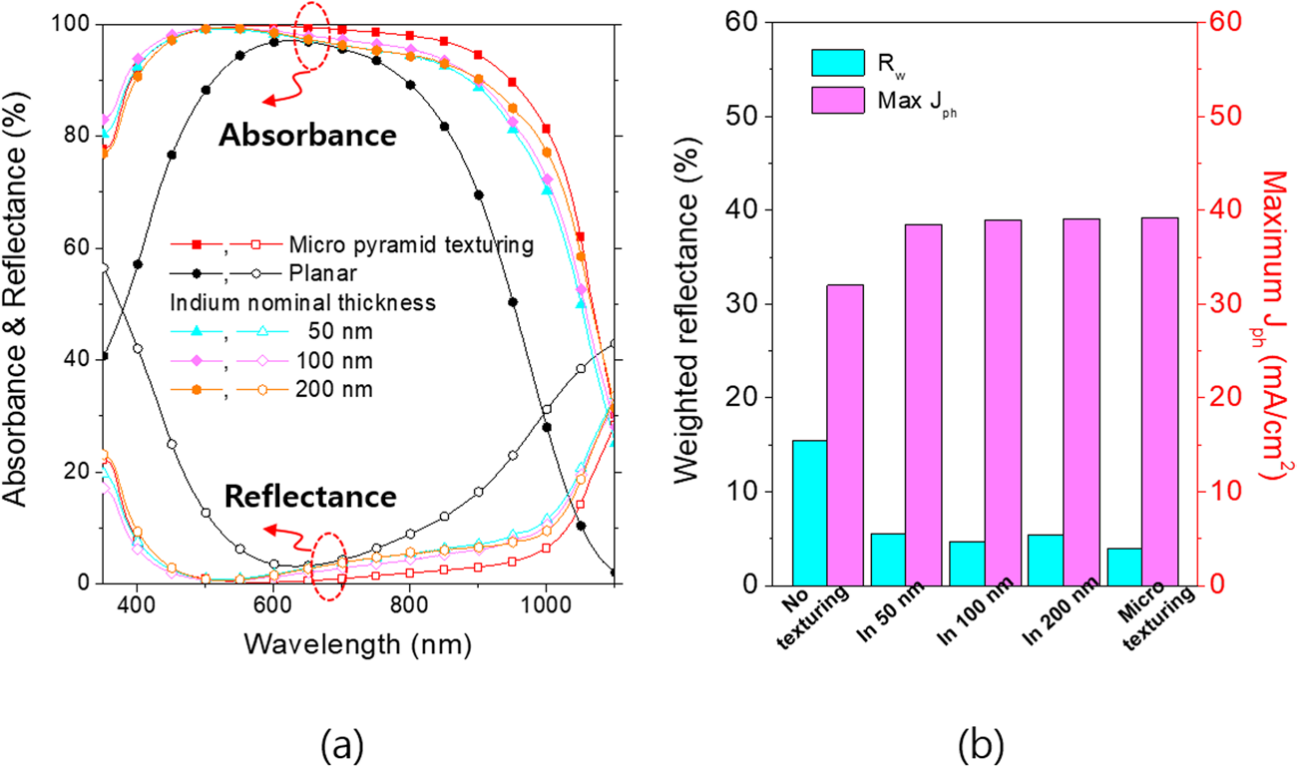
(**a**) Absorptances and reflectances of the crystalline Si thin foils (50 µm thickness) textured with the random Si nanohole arrays and coated with a antireflection coating of SiN_x_ (70 nm). For comparison, a planar Si thin foil without texturing and a textured one with conventional pyramids are compared. (**b**) Solar weighted reflectances and equivalent photocurrents of the various Si thin foils.

We introduced the random nanohole textures on the thin Si foils of a 50 µm thickness with antireflection coating of SiN_x_, and measured reflectances and absorptances. For the purpose of comparison, the optical properties of the planar and micro pyramid textured wafers are shown together. All the wafers textured with the random nanohole structures show broad range antireflections and in turn higher absorptances compared with the planar wafer. The micro pyramid textured wafers have slightly higher absorptances in the long wavelength rage above 700 nm. The maximum photocurrents converted from the measured absorptances of the thin foils were calculated assuming an internal quantum efficiency is 100% and presented in Fig. 6. The solar irradiation weighted reflectances are also given together. All the nanohole textured wafers exceed 90% of the ideal absorption limit (Lambertian limit, 42. 0 mA/cm^2^), resulting in the maximum photocurrents of 38.5 mA/cm^2^, 39.0 mA/cm^2^, 39.1 mA/cm^2^ for the indium masks of 50 nm, 100 nm and 200 nm nominal thicknesses, respectively^35^.

### LFC process and PERC Si solar cells

**Figure 7:**
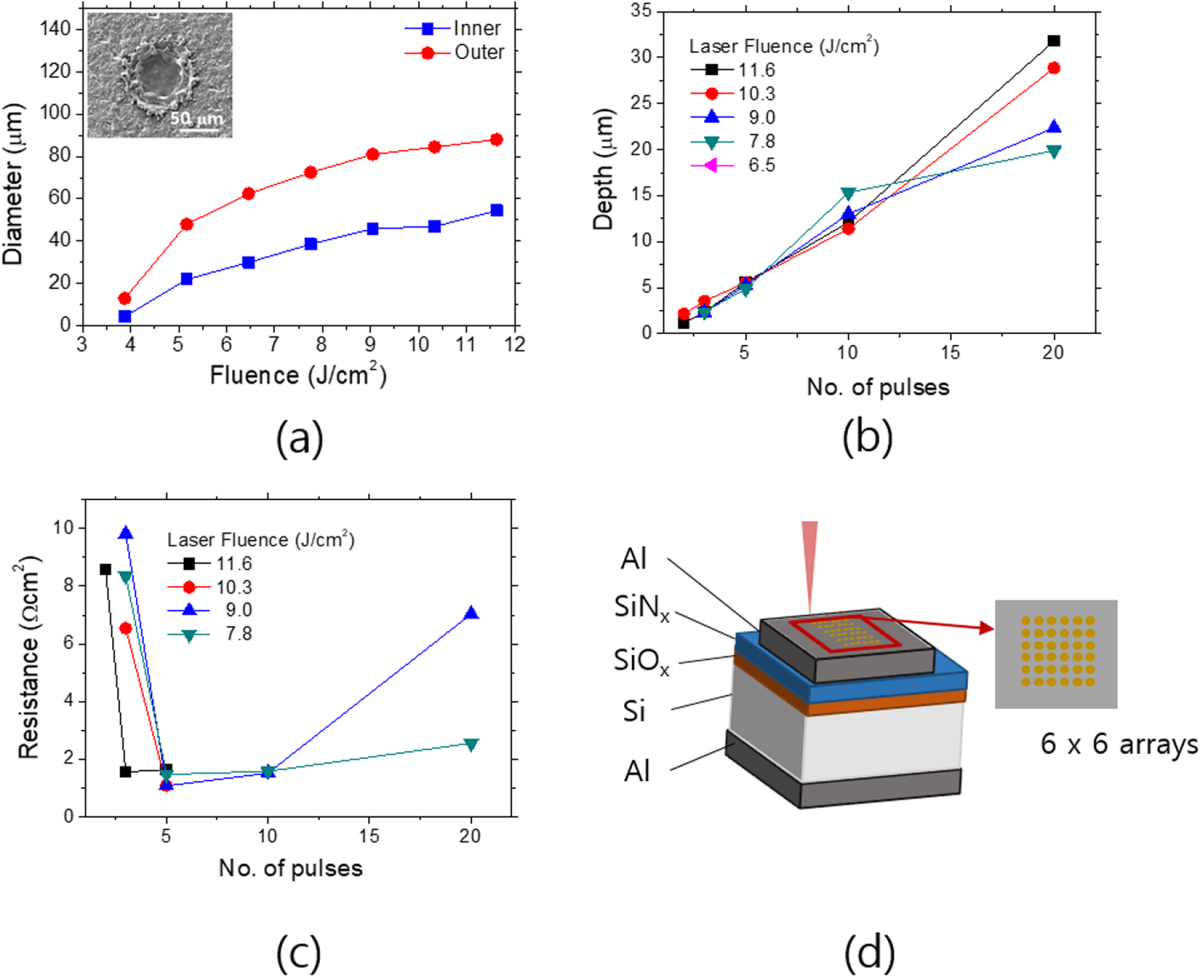
(**a**) Inner and outer diameters of LFC contact holes with varying laser fluences. The inset figure is the SEM image of the LFC contact hole processed with a 11.6 J/cm^2^ laser fluence and 5 laser pulses. (**b**) Depths of LFC contact holes with increasing the number of laser pulses and laser fluences. (**c**) The normalized resistances of the LFC contact hole arrays as a function of the laser pulses. (**d**) Schematic of the test structures for the resistance measurements. The pitch of the LFC contact hole arrays is set for 500 µm (6 × 6 arrays). The rear side has a full area Al electrode in ohmic contact with Si substrates. The passivation layers consist of SiO_x_ (20 nm)/SiN_x_ (80 nm) bilayers.

In the LFC process, a pulse laser is incident into the rear side aluminum layer and locally melts the aluminum to fire through the dielectric passivation layer resulting in the electrical contact with the Si base wafer. This process can be done at room temperature; thus, the wafer bowing can be avoided. For this reason, the LFC process is very compatible with the thin Si foil based solar cells^36^. For the successful adoption of the LFC process for the thin Si foils, the laser process parameters must be optimized in terms of the local contact hole depth and the contact resistance. The diameter of the contact hole can be adjusted by the laser fluence, and the depth by the number of the laser pulses^37^. The laser contact hole after the LFC process typically takes the shape of a crater. The diameter of the LFC contact hole is characterized by inner and outer diameters as shown in the incept figure of Fig. 7(a). The electrical contact between Al and Si is made through the inner contact hole. The laser intensity profile follows the Gaussian distribution; thus, the laser fluence increases the beam diameter in proportion to the laser fluence following the relationship of *d*^2^~log(*E*)^38^. The inner and outer diameters as a function of the laser fluence (*E*) with 5 laser pulses are presented in Fig. 7(a). Both of the diameters as a function of the laser fluence provide the good fit to the above model in a wide range of the laser fluences as shown in Fig. S2. The depths of the contact holes increase linearly with the number of the laser pulses irrespective of the laser fluence as shown in Fig. 7(b). The depths of the contact holes were determined by measuring the distance from the interface of Si/dielectric passivation layer to the dip of the contact holes using the 3-dimenional optical microscope. The cross-sectional profiles of the contact holes at selected laser processing conditions reveal the shape of the craters comprising the inner and outer rings as illustrated in Fig. S3. The depth of the contact hole must be much shallower than the wafer thickness to suppress the carrier recombination at the contact hole. At the same time, the contact resistance of the LFC contact needs to be minimized for suppression of a parasitic power loss. The contact resistances of the LFC contact holes with the test structure were analyzed with the varying laser fluences and the number of pulses as shown in Fig. 7(c). In the early stage of the laser pulses, the resistance is high, but above a threshold number of the laser pulses, the resistances exhibit the minimum in the range of 3~5 pulses depending on the laser fluences and increase again gradually with the laser pulses, which is attributed to the excessive ablation of Al. The bulk resistance in the Si wafers also contributes to the resistances determined above. In order to exclude the bulk contribution to the contact resistances, we fabricated the TLM (Transfer Length Measurement) test structures as illustrated in Fig. S4^39^. The extracted specific contact resistances of the LFC contact holes were all below 7 mΩcm^2^ for the laser fluences of 10.3, 11.6 J/cm^2^, We applied 10.3 J/cm^2^ and 5 laser pulses as the optimal laser processing conditions for the thin Si foils based PERC cell. At this condition, the inner diameter and depth of the LFC circular contact holes were 47 µm and 5.6 µm respectively with the specific contact resistance of 4.8 mΩcm^2^. The pitch between the LFC points was adjusted to be 700 µm. The depth of the LFC contact holes was much shallower than that of the industrial PERC contact holes which is usually tens of micrometer^40^; thus, our LFC scheme is very applicable to the thin Si foil based solar cells.

**Figure 8:**
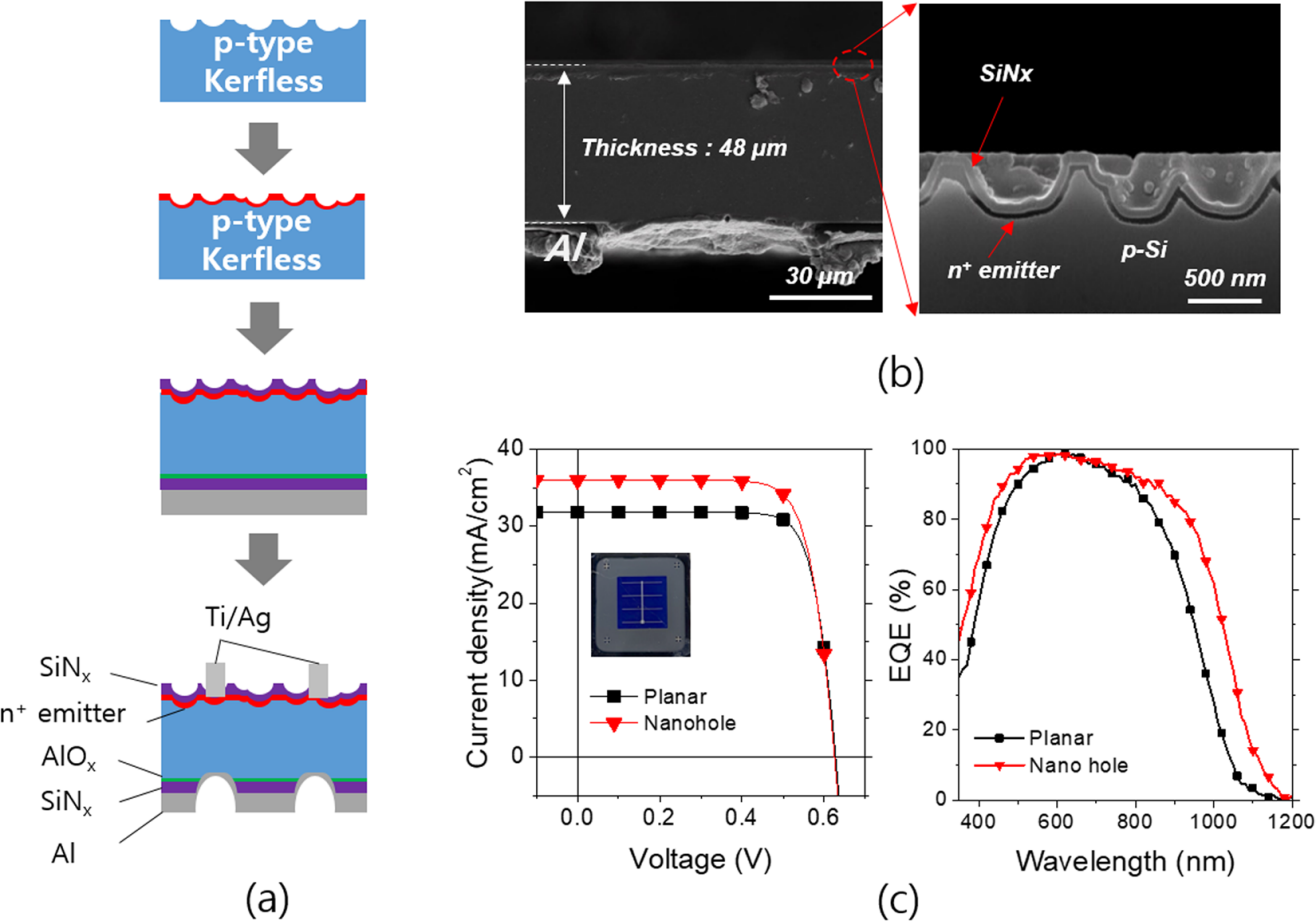
(**a**) Fabrication flow of PERC solar cells. (**b**) Cross-sectional SEM images of the PERC solar cell based on the Si thin foil of a 48 µm thickness (left) and the SiNx passivated emitter textured with the Si nanohole arrays (right). (**c**) Current-voltage characteristics of the PERC solar cells under illumination of a standard solar irradiation. EQE spectra of the PERC solar cells without texture (planar) and with nanohole arrays.

The Si thin foil of a 60 µm thickness was produced by the PIE technique, and the implantation damage was completely removed by surface etching of 6 µm at both sides as aforementioned. We started the PERC cell processing with the damage-removed Si thin foils. The random nano-hole structures were incorporated on the Si thin foils as depicted in Fig. 8. The indium islands of a 50 nm nominal thickness were chosen as the etch masks for the nanohole structure fabrication. The rear side of the thin foils was passivated with the bilayer dielectric layers of AlO_x_ (20 nm) and SiN_x_ (80 nm). The rear side local contacts were formed by the LFC process with square arrays of the point contacts. The cross-sectional SEM images of the LFC solar cell in Fig. 8(b) show the front side nanohole structures and the rear side LFC contact holes. The shallow LFC contact arrays of a few micrometer depth were formed by the optimal LFC process. The heavily doped emitter region, which was selectively etched by the HNA (HF:HNO_3_:Acetic acid) solution, is observed at the front side. Note that the passivation layer of SiN_x_ is coated conformably onto the nanohole structures. The planar solar cell without the nanohole structures was also fabricated for the purpose of the comparison. The performance parameters of the LFC solar cells were extracted from current-voltage characteristics in Fig. 8(c) and presented in Table 1. Whereas the planar LFC solar cell exhibited 15.6% of an efficiency, the nanohole textured cell showed 17.1%. This remarkable increase in the efficiency is due to the enhanced light trapping with incorporation of the nanohole texture; the short circuit current density (*J*_*sc*_) increased from 31.8 mA/cm^2^ to 35.9 mA/cm^2^. The external quantum efficiency (EQE) spectra of the nano texture solar cell show an EQE enhancement in a broad spectral range in comparison with the planar cell as seen in Fig. 8(c). In our previous report, we demonstrated the solar cells of a standard cell architecture or Al back surface field with the same Si thin foils used in this study and an efficiency of 15.2% was achieved^10^. The enhancement of the LFC PERC cell over the previous Al BSF cell is due to suppressed recombination and increased internal reflectance at the rear side, which are the main benefits we expect from the PERC solar cell architecture. As a result, the open circuit voltage and the short circuit current density improved in comparison with the Al BSF cell.

**Table 1:** Device performance parameters of the PERC solar cells based on the thin Si foils of a 48 µm produced by the PIE technique.

Cell type	Voc (mV)	Fill factor (%)	J_sc_ (mA/cm^2^)	Efficiency (%)
Planar	628	78.1	31.8	15.6
Random nanohole arrays	625	76.0	35.9	17.1

As shown in Fig. 1, the Si thin foils are bendable to have a bending radius of a few tens millimeter. We carried out a cyclic bending test for the Si thin foil solar cell and found the cell was bendable to have a 16 mm critical bending radius without significant performance degradation. More details are described in supplementary (Fig. S5).

Lastly, we have performed device simulations using PC1D to estimate the efficiency limit of our LFC PERC cell in Fig. S5. The simulation reveals that the current design of our LFC PERC cell is limited by the front and back surface recombination. The front surface recombination velocity (FSRV) and the back surface recombination velocity (BSRV) of our textured LFC PERC cell were 2 × 10^5^ cm/s and 300 cm/s, respectively. While keeping the current light trapping efficiency of the nanohole structures, we can expect to exceed an efficiency of over 19% if FSRV and BSRV decrease below 10^5^ cm/s, and 100 cm/s with slight reduction of series resistance (0.5 Ωcm^2^).

## Conclusions

In this study, we developed the fabrication process of the random nanohole arrays for effective light trapping especially in the Si thin foils. The low melting point metal of indium, which exhibit dewetting at room temperature, was used as nano scale etch masks. Two step etching process of directional dry and isotropic wet etching led to random Si nanohole structures of ellipsoids. The size and depth of the Si nanohole structures were adjusted by varying the indium nominal thickness and etching process time. The optimal nanohole structures achieved over 91% of the ideal light trapping performance. The optimally designed nanohole structures were introduced on the Si thin foils of a 48 um thickness and showed a comparable light trapping performance to the micro pyramid textures.

The LFC process for the Si thin foils was developed by tuning various laser processing parameters such as defocusing, laser pulse, and laser power. The shallow LFC contacts of a few micrometer depth and a low contact resistance were formed for the Si thin foils of a 48 um thickness. We prepared the kerfless Si thin foils by the proton implant exfoliation technique using a MeV proton accelerator. We introduced the Si nanohole structures on the kerfless Si thin foils, and the rear side local contact of Al back surface field was made by the LFC process. The optimally designed Si nanostructure and the LFC process led to 17.1% of an efficiency. Although the use of thinner Si foils not only allows for cost reduction of the c-Si solar cells but also provides new functionalities of flexibility and light-weight for c-Si solar cells, the industry has difficulty in adopting the thin foils for cell fabrication because of the thin cell processing issues. The proposed schemes of shallow texturing and rear side laser processing in this study would be well compatible with the thin cell processing and enable adoption of the thin foils for the low cost Si solar cells in the near-term future.

## Methods

### Fabrication of the ultrathin kerfless wafers by the PIE technique

We fabricated kerfless wafers by using a proton induced exfoliation (PIE) technique. For this study, the ultrathin (111) p-type silicon wafers were exfoliated from thick silicon wafers, which is termed as donor wafers, by using proton implantation with a 4 MV pelletron system at Korea Institute of Science and Technology (KIST). After cleaning the Si donor wafers (CZ, 2 Ω-cm) of (111) crystal orientation and a 300 μm thickness by standard RCA1 and RCA2 methods, the Si wafers were implanted with protons of 2.3 MeV at a dose of 1 × 10^17^/cm^2^. We subsequently annealed the donor wafers after ion implantation at 500 °C for 30 min to have the ultrathin wafers of a 58 μm thickness exfoliated. We successfully fabricated kerfless ultra-thin Si wafers of a 58 μm thickness with large area (5 cm × 5 cm) by the PIE process.

### Texturing process with random nanohole arrays

We studied nano texturing process that can be applicable to a large-area wafer. We fabricated the random ellipsoidal nanohole texturing by using chemical wet etch processing with dewetted indium metal as an etch mask. First, We a deposited silicon oxide film 200 nm as a buffer layer by Plasma enhanced chemical vapor deposition (CVD). We deposited the low melting point metal indium of 50 nm, 100 nm and 200 nm thicknesses to control the size of nanohole structures on the Si wafers. Deposition of indium metal by e-beam evaporation followed. Using the indium metal islands as an etch mask, a reactive ion etch (RIE) process was performed to etch the Si wafers, and the silicon oxide layer underneath was etched simultaneously. In this manner, the pattern of the indium islands is transferred to the silicon oxide layer. The RIE process was carried out using a mixed gas of CF_4_ (90%) and O_2_ (10%) for 15 min. The total gas pressure was fixed at 40 mTorr and the radio frequency (RF) power at 230 W. The silicon oxide buffer layer also serves as a protection layer for the Si wafers by preventing contamination during the RIE step and helps in lifting-off indium metal islands. In the next step, the indium metal islands were completely removed in a HCl solution. Lastly, the ellipsoidal nanohole structure are fabricated by isotropic etching of the silicon wafers with an HNP (HF:HNO_3_:H_3_PO_4_) solution. The shape of the ellipsoidal nanohole texturing was observed by FE-SEM (Nova-SEM).

### Optical reflectance and absorptance measurements

The front surface total reflectance and transmittance of ultrathin wafers with the ellipsoidal nano-hole structures was measured by a UV-Vis spectrophotometer (Perkin Elmer Lambda 35) with an integrating sphere in the wavelength range of 350 –1100 nm. The weighted reflectance (*R*_w_) is calculated by averaging the reflectance (*R*) over a wavelength range from 350 nm to 1100 nm with a weighting of a standard solar irradiation of AM 1.5 G (*I*) by the following equation.

1 $${R}_{w}=\frac{{\int }_{350\,nm}^{1100\,nm}R(\lambda )I(\lambda )d\lambda }{{\int }_{350\,nm}^{1100\,nm}I(\lambda )d\lambda }$$

The absorptances of the ultrathin Si wafers of a 50 µm were determined by total reflectances and transmittances using the integrating sphere in the same wavelength range. The equivalent maximum photon current (*J*_*ph*_) is calculated by assuming all the absorbed photons are converted into the electricity over the standard solar irradiation using the following equation.

2 $${J}_{ph}={\int }_{350\,nm}^{1100\,nm}\frac{q}{hc}\lambda A(\lambda )I(\lambda )d\lambda $$

where *A* is the absorptances, *q* is the unit charge, *h* is the Planck constant and *c* is the light speed constant.

### Cell fabrication process

In order to improve the efficiencies of the solar cells, we modified the PERC architecture of a standard structure by introducing nano textures at a front side and a passivation layer combined with alumina oxide (Al_2_O_3_) and silicon nitride (SiN_x_) layer at a rear side. As for the optical spacer, the passivation layer of SiN_x_ was chosen to increase an internal reflectance at the rear side of a Si wafer. We fabricated solar cell device based on kerfless wafer by using a proton induced exfoliation (PIE) technique. After cleaning the Si donor wafers (CZ, 2 Ω-cm, 1 cm × 1 cm) of a (111) crystal orientation and a 300 µm thickness by standard RCA1 and RCA2 methods, the Si wafers were implanted with protons of a 2.3 MeV acceleration energy and at a dose of 1 × 10^17^/cm^2^. We subsequently annealed the donor wafers after ion implantation at 500 °C for 30 min to exfoliate the ultrathin wafers below a thickness of 58 µm. The fabrication process of a high efficiency PERC (passivated and rear cell) solar cell is as follows in 12 steps: 1) kerfless wafering by using a PIE technique and cleaning of cleaved wafers by standard organic cleaning process and RCA1 and RCA2, 2) annealing of kerfless wafers in N_2_ at 900 °C for 10 min in the tube furnace, 3) damage removal etching in a HNA acid solution and subsequently etched by varying a removed thickness for minority carrier lifetime recovery, 4) A low cost doping method, POCl_3_ tube furnace, has been used in this case at 830 °C for 30 min (drive-in), 5) The Si substrates were rinsed in 25 wt% HF solution for a few minutes to remove the PSG (Phosphorous silicone glass), followed by deionized water rinse for several minutes, 6) Al_2_O_3_ (Aluminum oxide) 20 nm and SiNx (Silicon nitride) 80 nm deposition at rear side for formation of passivation layer by ALD and PECVD, 7) Al deposition at rear side on the passivation layer for back electrodes of a 2 μm by e-beam evaporation, 8) deposition of Ag front electrodes of a 2 μm thickness with the insertion of Ti buffer layer as adhesion layer by e-beam evaporation, 9) PECVD SiN_x_ 80 nm deposition at 400 °C, 10) LFC (laser fired contact) process at the rear side using a Nd:YVO_4_ pulsed laser with 1064 nm wavelength, The frequency of the q-switch can be chosen between 10 kHz to 100 kHz. The Nd:YVO_4_ rod is pumped by a single diode. The diode current can be fixed at 31 A, 11) edge isolation, and lastly 12) forming gas annealing at 400 °C for 40 min in the tube furnace to ensure good Ohmic contacts.

### Cell performance parameter measurements

The photovoltaic conversion efficiency and external quantum efficiency of these solar cells were measured by Solar simulator at a light intensity of 100 mW/cm^2^ of a standard AM 1.5 G irradiation and varying the wavelength of the incident monochromatic light from 350 nm to1200 nm respectively. As a result, the highest efficiency of 17.1% is achieved at a 48 μm thickness. Note that PERC cell has the highest photocurrent of 35.9 mA/cm^2^ among cells. This is attributed to the increased internal reflectance of the rear side by introducing passivation layer of a 100 nm thickness. As a thickness of the Si wafers decreases more photons penetrate into the rear side; thus, higher internal reflectance at the rear side is beneficial for thinner Si wafers.

## Supplementary information

Supplementary Document
