# Supplementary material for: Random nanohole arrays and its application to crystalline Si thin foils produced by proton induced exfoliation for solar cells
Source: Sci Rep. 2019 Dec 24;9:19736. doi: 10.1038/s41598-019-56210-7 (PMC6930296; doi:10.1038/s41598-019-56210-7)
Supplement: Supplementary file 1 — Supplementary Document [file 41598_2019_56210_MOESM1_ESM.docx]

**Supplementary materials**

**Random nanohole arrays and its application to crystalline Si thin foils produced by proton induced exfoliation for solar cells**

Hyeon-Seung Lee^1,2^, Jae Myeong Choi^1,2^, Beomsic Jung^1,2^, Joonkon Kim^3^, Jonghan Song^3^, Doo Seok Jeong^4^, Jong-Keuk Park^1^, Won Mok Kim^1^, Doh-Kwon Lee^5^, Taek Sung Lee^1^, Wook Seong Lee^1^, Kyeong-Seok Lee^1^, Byeong-Kwon Ju^2^, Inho Kim^1^

*^1^Center for Electronic Materials, Korea Institute of Science and Technology, Seongbuk-gu, Seoul 02792, Republic of Korea*

*^2^ School of Electrical Engineering, Korea University, Seoul 02841, Republic of Korea*

*^3^Advanced Analysis Center, Korea Institute of Science and Technology, Seongbuk-gu, Seoul 02792, Republic of Korea*

*^4\^Division of Materials Science and Engineering, Hanyang University, Seoul 04763, Republic of Korea*

*^5^Photo-electronic Hybrids Research Center, Korea Institute of Science and Technology, Seongbuk-gu, Seoul 02792, Republic of Korea*

In order to provide a design guide for further optimization of the random ellipsoidal nanohole arrays in our solar cells, we performed optical simulations assuming them as periodic nano hole arrays in a square lattice. We carried out optical simulations by using a commercial rigorous coupled wave analysis (RCWA) package (AlSoft DiffractMod). The shape of the nanostructure was assumed to be an elongated hemi-ellipsoid in a vertical direction; the radii in x- and y-directions are the same and the radius in the z-direction is larger. The fractional area of the ellipsoidal nano-holes at the surface of the Si wafer is fixed to be 78 % for all the calculations, and an antireflection coating (ARC) layer with a refractive index of 1.9 is assumed to cover the nanohole arrays. The Si wafer was set to be semi-infinite for the sake of convenience. The reflectances were calculated as a function of the depth and diameter of the Si hemi-ellipsoid. Assuming except the reflected light, all the incident photons are absorbed, we calculated the equivalent photocurrents in two wavelength ranges of short (350 nm ~ 700 nm) and long (700 ~ 1200 nm) ranges and shown in Figure S1.


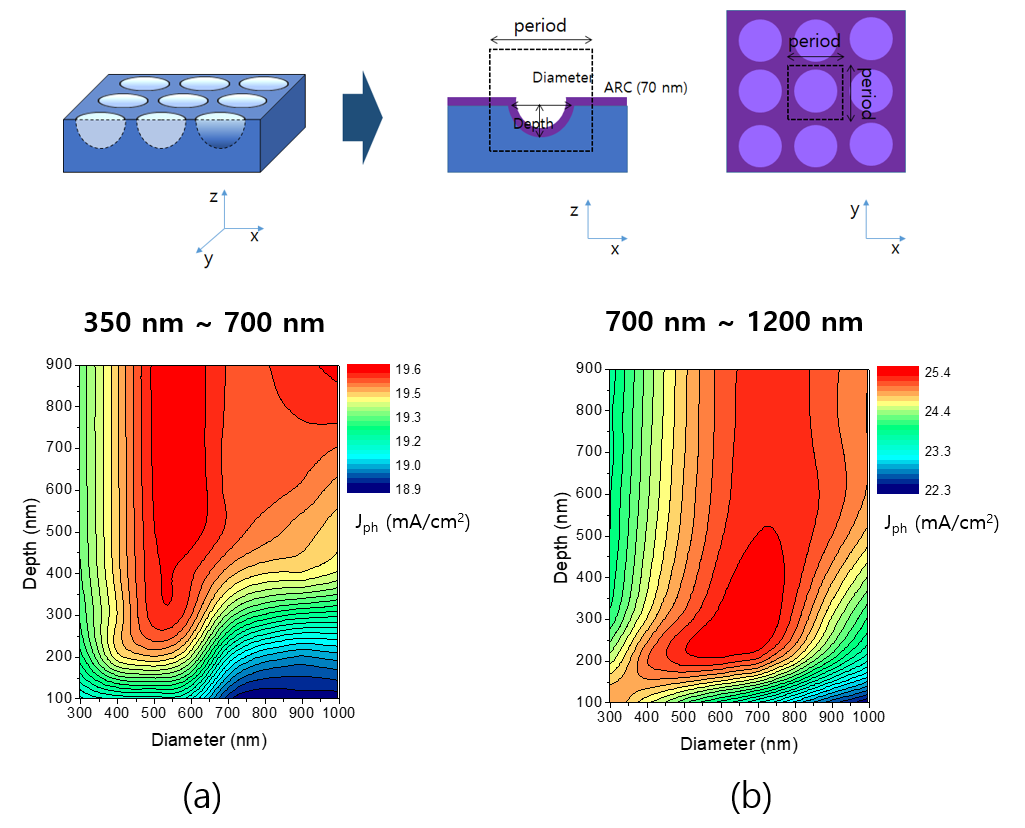


Figure S1. Simulated photocurrents (J_ph_) by a RCWA method as a function of depths and diameters of ellipsoidal nanohole arrays in two different wavelength ranges: (a) short wavelength range, (b) long wavelength rage.


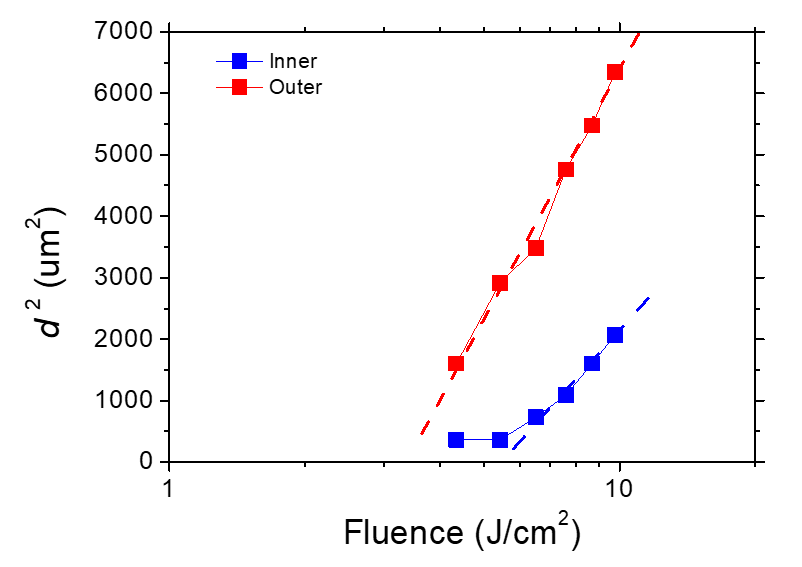


Figure S2. *d^2^* vs. log (fluence) plot of Figure 7 (a) in the main manuscript.


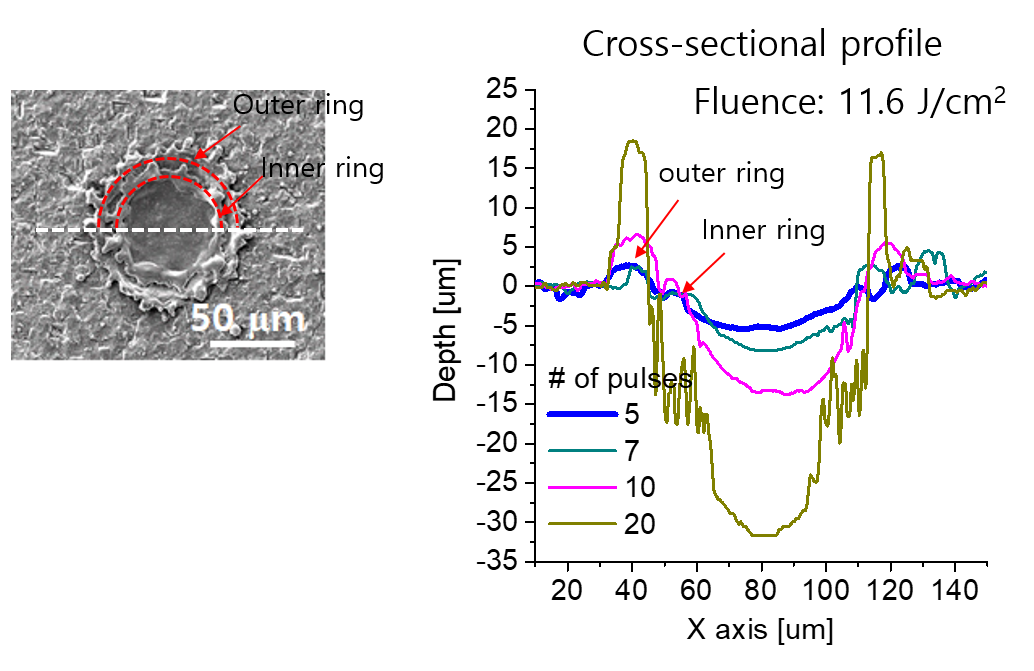


Figure S3. Cross-sectional profiles of the LFC contact holes measured by 3-dimensional optical microscope (right). The double (inner and outer) rings in the SEM image (left) are clearly observed in the cross-sectional profiles. The inner and outer rings are denoted by the arrows.

The schematic of a TLM test structure is illustrated as below in Figure S4. The width and length (W) of the aluminum pad are 400 µm and 1.0 mm, respectively. The distance (d) of the neighboring pads is varied form 500 µm to 3500 µm. The LFC contact hole arrays with a pitch of 1000 µm were processed at each pad. The contact resistance of the Al contact pad is determined by measuring the resistance and using the following equation (S1). The specific contact resistance ($\rho_{c}$) of each contact hole is determined from the equation by normalization to the total contact hole area ($n\cdot\pi r^{2}$) at each pad, where n is the number of the contact holes. *R_sh_* is the sheet resistance of the wafer, *R_T_* is the resistance between neighboring pads, and *R_c_* is the contact resistance.

$R_{T}= \frac{R_{sh}}{W}d+2R_{c}$ (S1)

$\rho_{c}=\frac{R_{c}}{n\cdot\pi r^{2}}$ (S2)


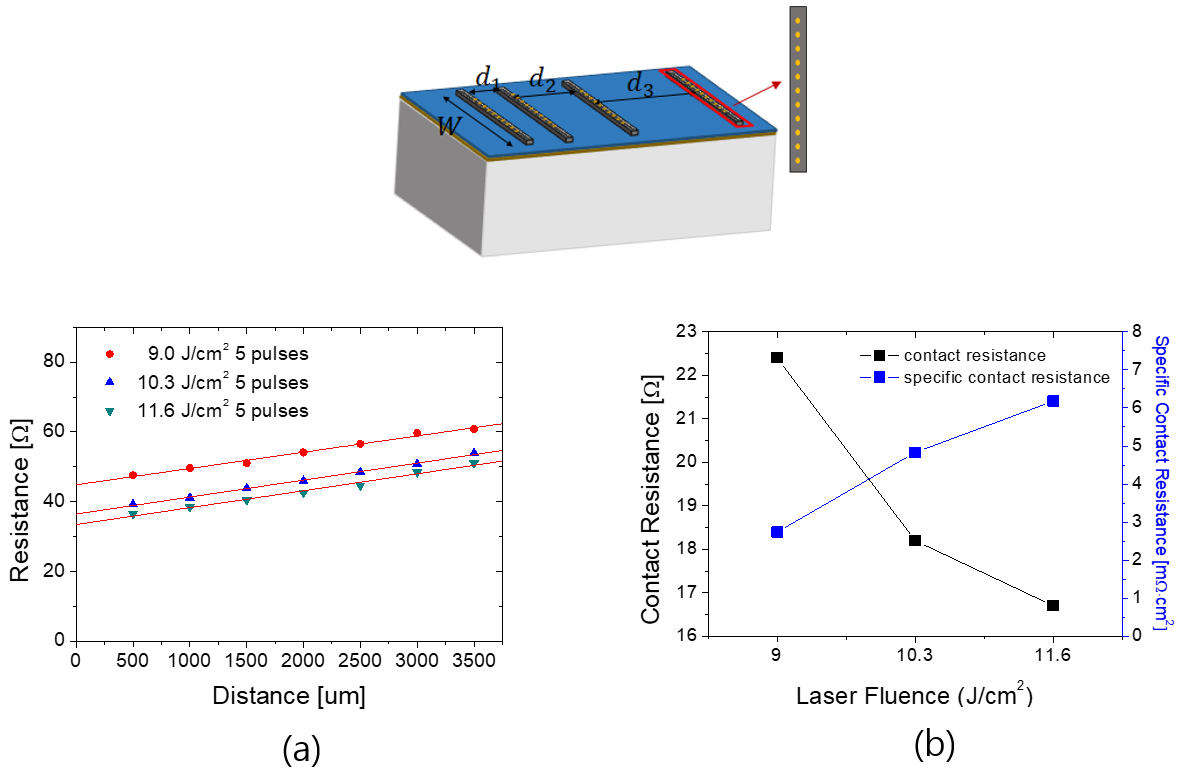


Figure S4. Schematic of a TLM test structure (top). (a) TLM measurement results with varying laser pulses. (b) Contact resistances and specific contact resistances with varying laser pulses determined by the TLM method.

We performed a cyclic bending test of the ultra-thin Si solar cell based on the kerfless wafer of a 48 um produced by the PIE technique. We placed the ultra-thin Si solar cell on plastic pipes of various radius from 45 mm to 13 mm and carried out 100 cyclic bending tests manually. After each 100 times cyclic bending test, the cell efficiency was measured on a planar plate under a standard solar irradiation condition. The cell efficiency showed slight fluctuations which are considered to come from testing condition issues, not from cell performance degradation. We think these variations might come from the testing instability such as the probe contact or fluctuations of the illumination light intensity. The ultra-thin solar cell survived the last bending of a 16 mm radius, and it was broken at a 13 mm bending radius.


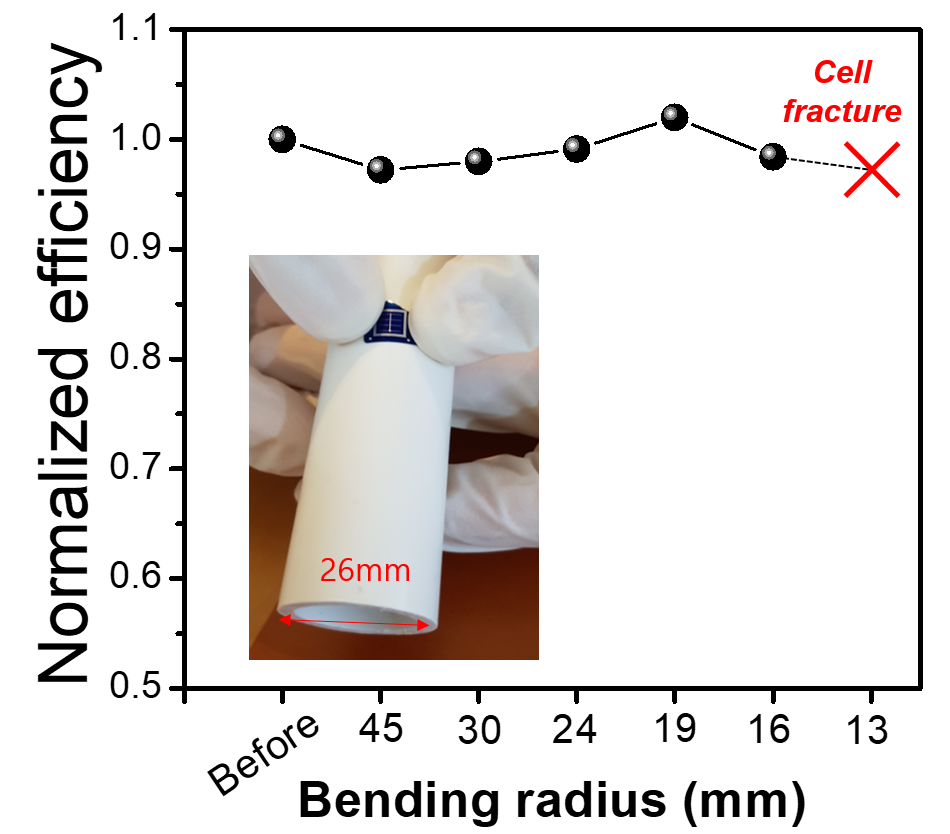


Figure S5. Normalized efficiency of the ultra-thin Si solar cell based on the kerfless wafer of a 48 um produced by the PIE technique after the cyclic bending test. The cell efficiency was measured after each 100 times cyclic bending test with various bending radius. The cell efficiency was normalized to that of the cell before a bending test.

We performed the PC1D simulations to find the front and back surface recombination velocities (FRSV, BSRV) of our current LFC PERC cells. We varied FRSV and BSRV to find the best fit of the simulated EQE curves to the experimental curve while keeping other parameters fixed such as the bulk lifetime, series resistance, and emitter doping concentration. The emitter resistance was 150 Ω/□ with a junction depth of 0.3 µm, and the series resistance was 1.3 Ωcm^2^. Also, the rear reflectance of the cell was set for 95 % specular. The parameters for the best fit were organized in Table S1. In order to the estimate the efficiency limit of our LFC PERC cell, we further varied the FSRV and BSRV values to obtain the efficiency greater than 19.1 %.


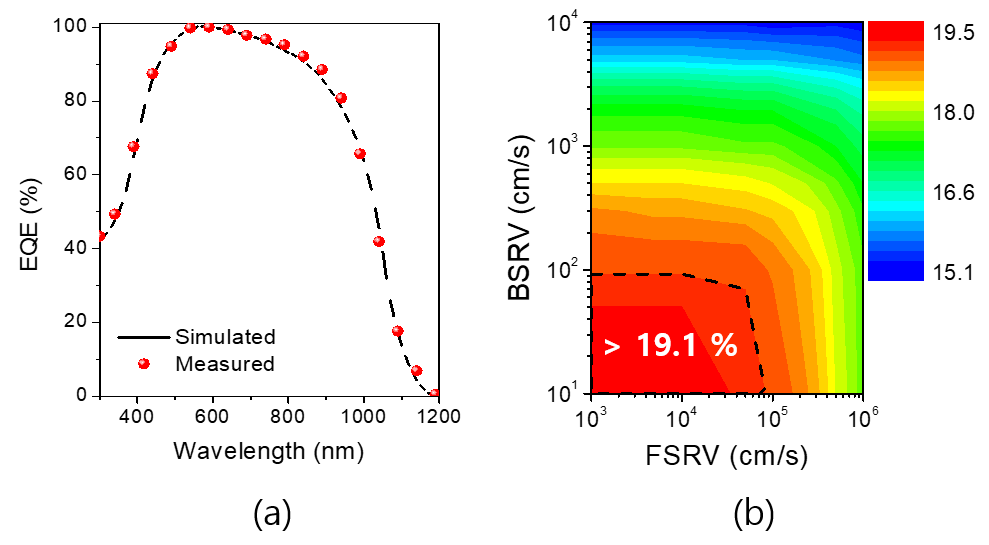


Figure S6. (a) EQE curve for the PERC solar cell textured with random Si nanohole arrays in the manuscript and fitted one simulated by PC1D. The material parameters used in the simulation is summarized in Table S1. (b) Efficiency mapping of the PERC solar cells as a function of front surface recombination velocity (FSRV) and back surface recombination velocity (BSRV).

Table S1. Device parameters fitted to the PERC solar cell (a 17.1 % efficiency cell) in this study.

|  | **Measured** | **Simulated** |
| --- | --- | --- |
| Efficiency (%) | 17.1 | 17.4 |
| Fill factor (%) | 76.0 | 76.4 |
| Voc (V) | 625 | 622 |
| EQE J_sc_ (mA/cm^2^) | 36.8 | 36.6 |
| R_s_ (Ohmcm^2^) | 1.1 | 1.3 |
| FSRV (cm/s) |  | 1.5x10^5^ |
| BSRV (cm/s) |  | 300 |
| Bulk lifetime (µs) |  | 200 |
